# Supplementary material for: Hearing loss and physical function in the general population: A cross-sectional study
Source: PLoS One. 2022 Oct 7;17(10):e0275877. doi: 10.1371/journal.pone.0275877 (PMC9544020; doi:10.1371/journal.pone.0275877)
Supplement: S1 Table — (DOCX) [file pone.0275877.s001.docx]

**S1 Table. Crude odds ratios for association between hearing loss and each variable**

| Variable | Male | | | Female | | |
| --- | --- | --- | --- | --- | --- | --- |
|  | n | OR [95% CI] | *p* value | n | OR [95% CI] | *p* value |
| Age, years | 2694 | 1.111 [1.099-1.124] | <0.001 | 2072 | 1.097 [1.081-1.113] | <0.001 |
| Handgrip strength, kg | 2601 | 0.964 [0.948-0.981] | <0.001 | 1971 | 0.894 [0.861-0.928] | <0.001 |
| VC, L | 1014 | 0.337 [0.265-0.429] | <0.001 | 669 | 0.204 [0.126-0.330] | <0.001 |
| FEV_1_, L | 1014 | 0.271 [0.208-0.353] | <0.001 | 669 | 0.121 [0.068-0.213] | <0.001 |
| Hypertension, yes/no | 1153/1541 | 2.740 [2.205-3.406] | <0.001 | 581/1491 | 2.340 [1.734-3.157] | <0.001 |
| Diabetes, yes/no | 217/2359 | 2.359 [1.720-3.236] | <0.001 | 69/1847 | 2.137 [1.146-3.982] | 0.017 |
| Current smoking, yes/no | 860/1710 | 0.762 [0.603-0.963] | 0.023 | 208/1704 | 0.822 [0.495-1.367] | 0.451 |
| Alcohol consumption ^a)^, yes/no | 804/1757 | 1.498 [1.201-1.867] | <0.001 | 173/1731 | 0.704 [0.392-1.266] | 0.241 |

Abbreviations: OR, odds ratio; 95% CI, 95% confidence interval; VC, vital capacity; FEV_1_, forced expiratory volume in one second

Hearing loss was defined as hearing threshold of > 30 dB at 1 kHz and/or > 40 dB at 4 kHz in either ear with pure-tone audiometry.

OR with 95% CI and *p* value were calculated using logistic regression analysis.

a) Defined as drinking everyday
