# Supplementary material for: Hearing loss and physical function in the general population: A cross-sectional study
Source: PLoS One. 2022 Oct 7;17(10):e0275877. doi: 10.1371/journal.pone.0275877 (PMC9544020; doi:10.1371/journal.pone.0275877)
Supplement: S2 Table — (DOCX) [file pone.0275877.s002.docx]

**S2 Table. Characteristics of study subjects in high and low physical function groups**

1. Handgrip strength (n=4572)

|  | | Handgrip strength | | | | |  |
| --- | --- | --- | --- | --- | --- | --- | --- |
| Variable | | Low group (< median) | |  | High group (≥ median) | | *p* value |
|  | | n |  |  | n |  |  |
| Sex, male, n (%) | | 2249 | 1288 (57.3) |  | 2323 | 1313 (56.5) | 0.610 |
| Age, years, mean, SD | | 2249 | 49.2, 14.8 |  | 2323 | 46.0, 12.6 | <0.001 |
| BMI, kg/m^2^, mean, SD | | 2249 | 23.2, 3.9 |  | 2323 | 24.3, 4.1 | <0.001 |
| Hearing loss ^a)^, n (%) | | 2249 | 357 (15.9) |  | 2323 | 223 (9.6) | <0.001 |
| Comorbidities, n (%) | |  |  |  |  |  |  |
|  | Hypertension | 2249 | 833 (37.0) |  | 2323 | 819 (35.3) | 0.210 |
|  | Diabetes | 2121 | 152 (7.2) |  | 2190 | 118 (5.4) | 0.016 |
|  | Dyslipidemia | 2204 | 1010 (45.8) |  | 2281 | 1071 (47.0) | 0.449 |
| Medical history, n (%) | |  |  |  |  |  |  |
|  | Stroke | 2117 | 38 (1.8) |  | 2184 | 29 (1.3) | 0.216 |
|  | Heart disease | 2118 | 74 (3.5) |  | 2184 | 47 (2.2) | 0.008 |

b) VC (n=1683)

|  | | VC | | | | |  | |
| --- | --- | --- | --- | --- | --- | --- | --- | --- |
| Variable | | Low group (< median) | |  | High group (≥ median) | | *p* value | |
|  | | n |  |  | n |  |  |  |
| Sex, male, n (%) | | 837 | 506 (60.5) |  | 846 | 508 (60.0) | 0.865 |  |
| Age, years, mean, SD | | 837 | 60.8, 10.6 |  | 846 | 50.3, 10.5 | <0.001 |  |
| BMI, kg/m^2^, mean, SD | | 837 | 23.9, 3.7 |  | 846 | 23.7, 3.5 | 0.152 |  |
| Hearing loss ^a)^, n (%) | | 837 | 251 (30.0) |  | 846 | 110 (13.0) | <0.001 |  |
| Comorbidities, n (%) | |  |  |  |  |  |  |  |
|  | Hypertension | 837 | 424 (50.7) |  | 846 | 269 (31.8) | <0.001 |  |
|  | Diabetes | 837 | 94 (11.2) |  | 846 | 37 (4.4) | <0.001 |  |
|  | Dyslipidemia | 837 | 478 (57.1) |  | 846 | 387 (45.7) | <0.001 |  |
| Medical history, n (%) | |  |  |  |  |  |  |  |
|  | Stroke | 835 | 27 (3.2) |  | 845 | 13 (1.5) | 0.023 |  |
|  | Heart disease | 837 | 45 (5.4) |  | 845 | 26 (3.1) | 0.019 |  |

c) FEV_1_ (n=1683)

|  | | FEV1 | | | | |  |
| --- | --- | --- | --- | --- | --- | --- | --- |
| Variable | | Low group (< median) | |  | High group (≥ median) | | *p* value |
|  | | n |  |  | n |  |  |
| Sex, male, n (%) | | 851 | 512 (60.2) |  | 832 | 502 (60.3) | 0.943 |
| Age, years, mean, SD | | 851 | 62.1 (9.5) |  | 832 | 48.8 (10.0) | <0.001 |
| BMI, kg/m^2^, mean, SD | | 851 | 23.9 (3.6) |  | 832 | 23.7 (3.6) | 0.175 |
| Hearing loss ^a)^, n (%) | | 851 | 268 (31.5) |  | 832 | 93 (11.2) | <0.001 |
| Comorbidities, n (%) | |  |  |  |  |  |  |
|  | Hypertension | 851 | 446 (52.4) |  | 832 | 247 (29.7) | <0.001 |
|  | Diabetes | 851 | 96 (11.3) |  | 832 | 35 (4.2) | <0.001 |
|  | Dyslipidemia | 851 | 487 (57.2) |  | 832 | 378 (45.4) | <0.001 |
| Medical history, n (%) | |  |  |  |  |  |  |
|  | Stroke | 849 | 31 (3.7) |  | 831 | 9 (1.1) | <0.001 |
|  | Heart disease | 851 | 49 (5.8) |  | 831 | 22 (2.6) | 0.002 |

Abbreviations: SD, standard deviation; BMI; body mass index; VC, vital capacity; FEV_1_, forced expiratory volume in one second

The subjects were divided into two groups by sex-specific median of handgrip strength, VC, and FEV_1_: ≥ sex-specific median as high group, < sex-specific median as low group.

*P* value was calculated using t-test for continuous variables and chi-square test for categorical variables.

a) Defined as hearing threshold of > 30 dB at 1 kHz and/or > 40 dB at 4 kHz in either ear with pure-tone audiometry
