# Supplementary material for: Hearing loss and physical function in the general population: A cross-sectional study
Source: PLoS One. 2022 Oct 7;17(10):e0275877. doi: 10.1371/journal.pone.0275877 (PMC9544020; doi:10.1371/journal.pone.0275877)

S3 Fig. Histograms of physical function by sex

a) Handgrip strength

Male

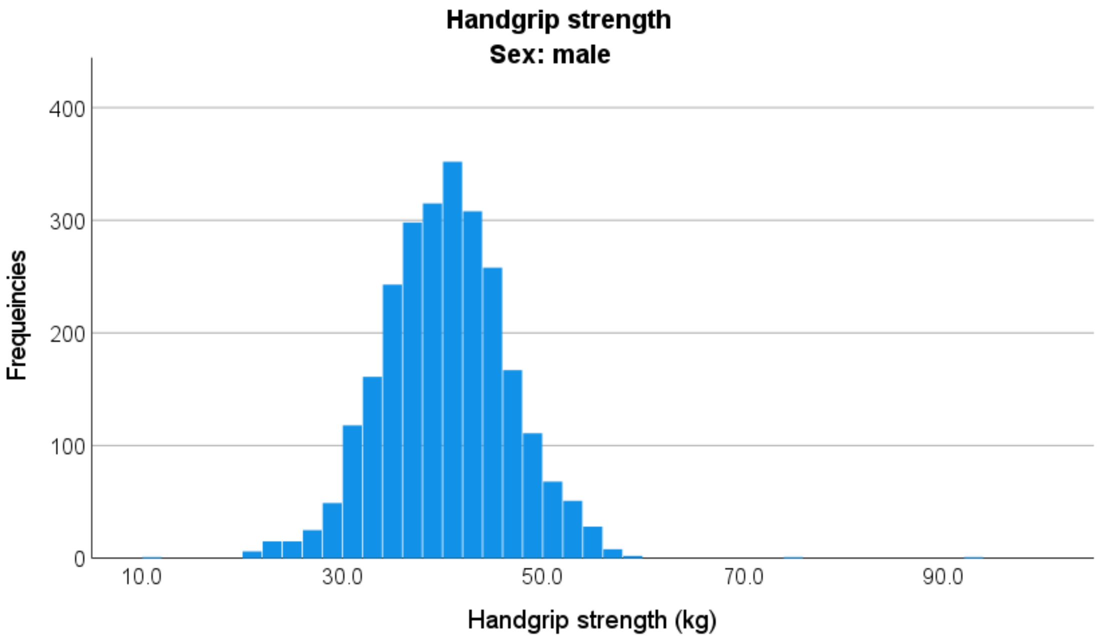

Female

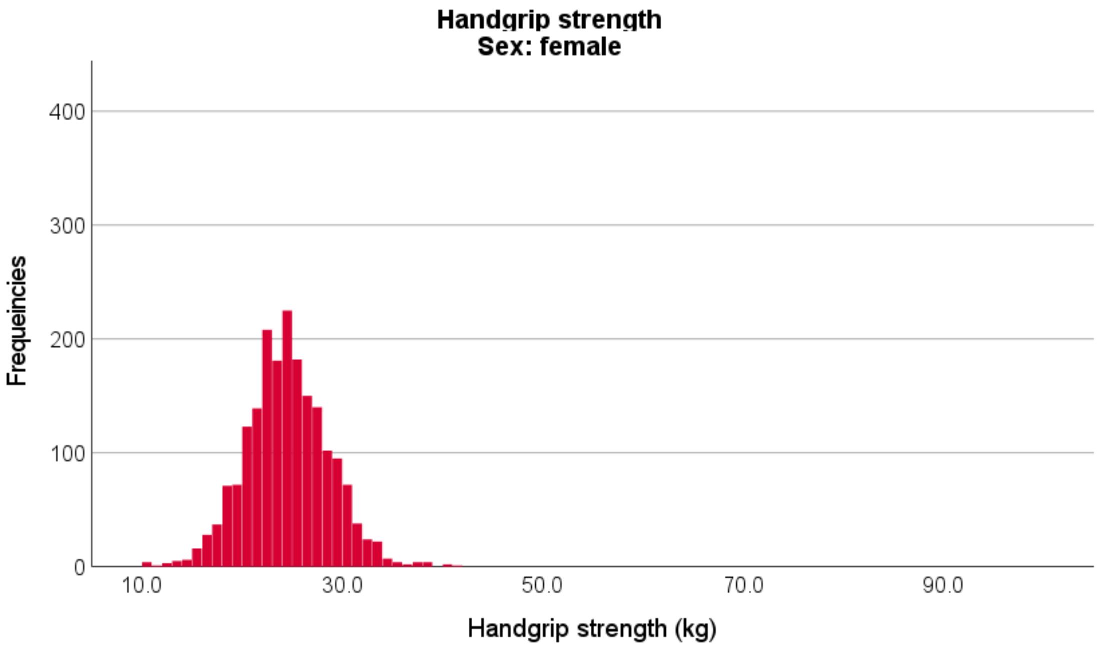

b) VC (vital capacity)

Male

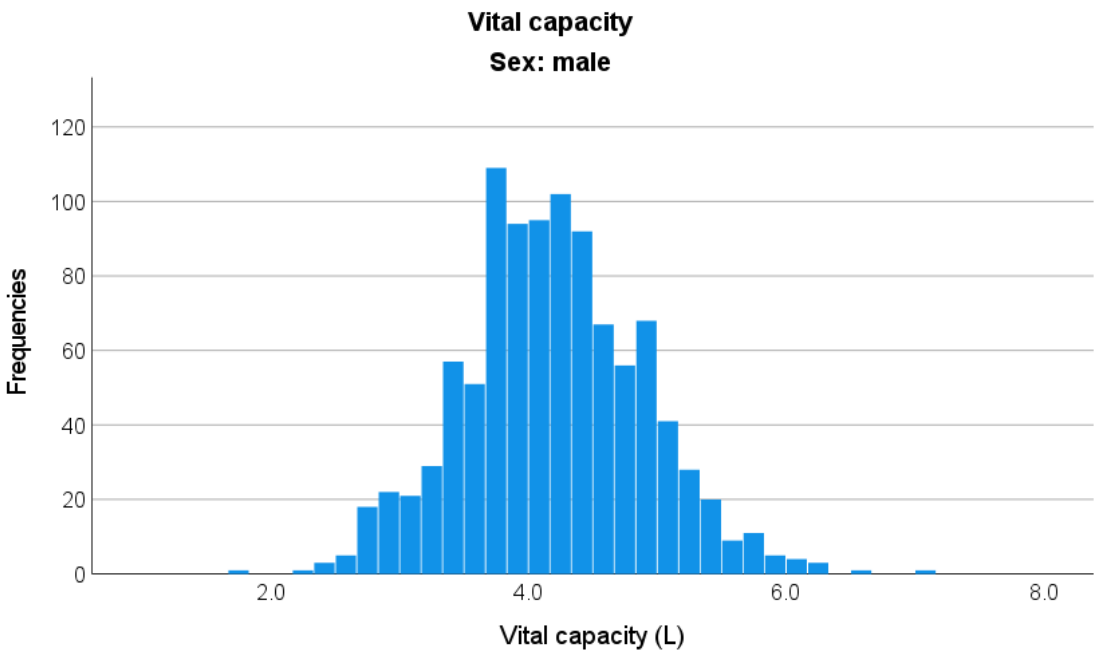

Female

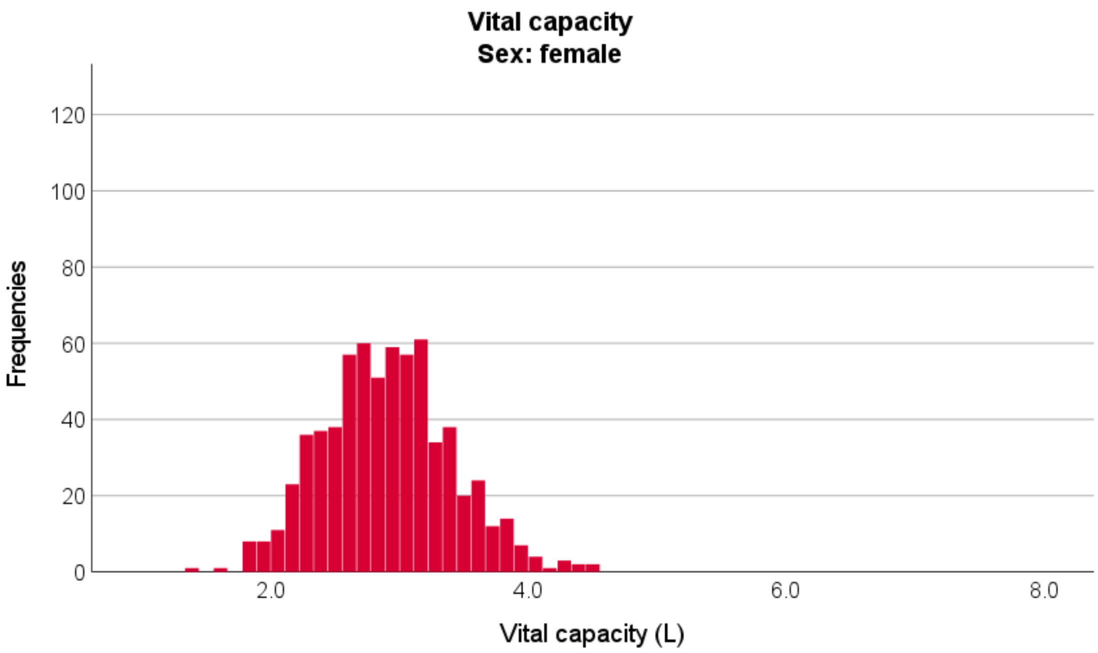

c) FEV_1 (forced expiratory volume in 1 second)

Male

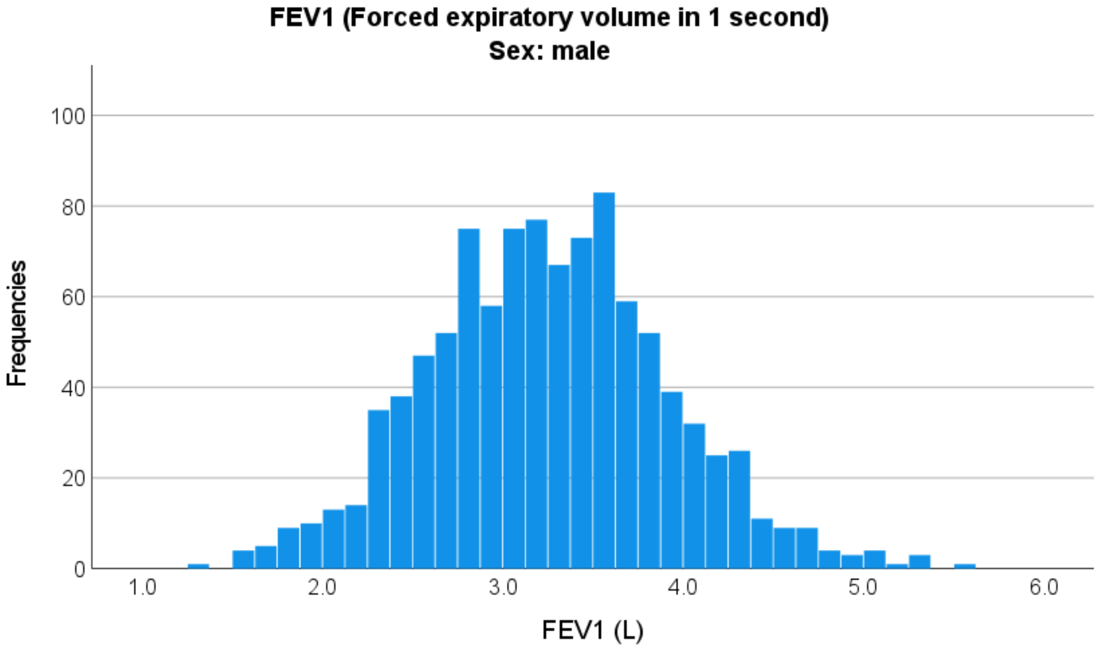

Female

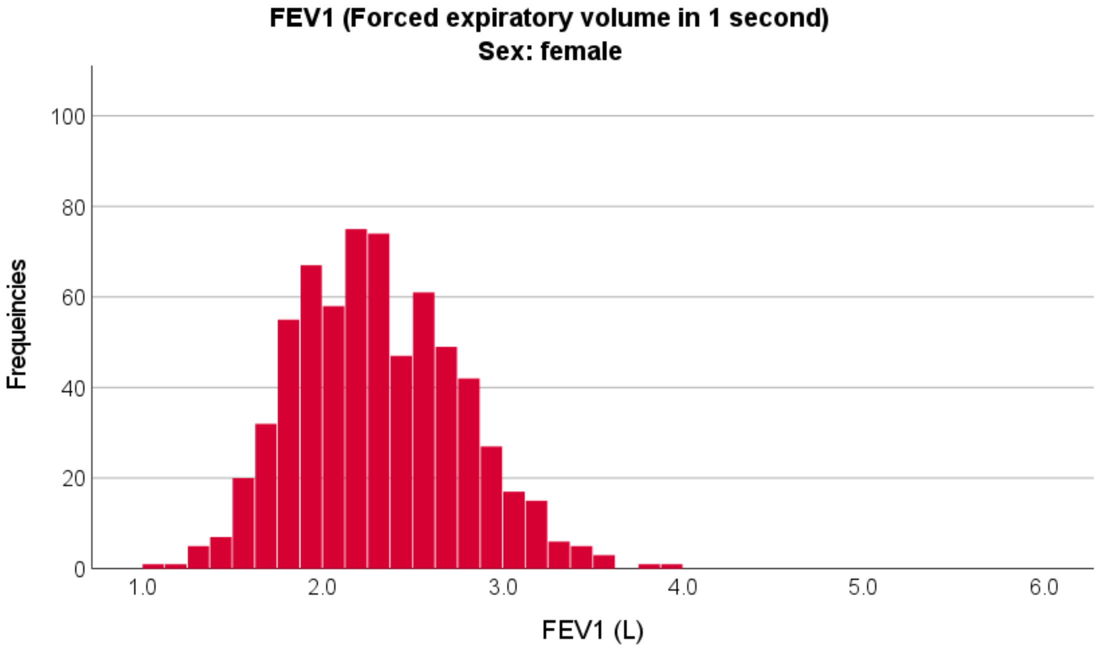

Supplement: S1 Fig — (PDF) [file pone.0275877.s003.pdf]
