# Supplementary material for: Hearing loss and physical function in the general population: A cross-sectional study
Source: PLoS One. 2022 Oct 7;17(10):e0275877. doi: 10.1371/journal.pone.0275877 (PMC9544020; doi:10.1371/journal.pone.0275877)
Supplement: Attachment — Submitted filename: Response to Reviewers.docx [file pone.0275877.s006.docx]

**Response to Reviewers**

Thank you very much for providing valuable comments to improve the manuscript. We revised according to the reviewer’s comments.

**Reviewer #1:** Thank you for the revised manuscript! I still have a few minor comments:

**Comment 1:** Please report results of male participants in the abstract.

**Response 1:** The result of male participants was stated in the abstract. (ll. 44)

**Comment 2:** ll. 245-252: As suggested, previous Table 2 was moved to the supplementary material. At the same time, the description of this table was not changed and covers an entire paragraph in the results section. Maybe you should move the table back to the results section if you want to describe the results more prominently. Otherwise, I would suggest referencing S2 at an appropriate position without describing the whole table.

**Response 2:** A shortened statement to refer previous S2 Table was re-positioned in ll. 239-242 (new number S4 Table). Supplemental files were re-numbered according to the order of appearance in text.

**Comment 3:** ll. 264-271: This paragraph explains why all further analyses were stratified by sex. Yet, the analyses before this paragraph were also stratified by sex. I suggest moving the content of this paragraph to the description of statistical analyses in the methods section.

**Response 3:** Thank you very much for mentioning this point. We agree to shift this part to Methods (ll. 174-178).

**Comment 4:** A reference for S1 and S4 is missing in the manuscript.

**Response 4:** A reference for previous S1 (ll. 234; new number S3) and previous S4 (ll. 215; new number S2) was added.

**Reviewer #2:** Thank you for addressing my comments. Some additional comments:

**Comment 1:** Introduction. The introduction should focus more on the association between physical function and hearing loss and summarize the gaps of previous studies looking at this association. Finally, you should mention how this study addresses the gaps in previous literature.

**Response 1:** Thank you for pointing this crucial point. We agree previous writing of introduction does not flow well into the study objective. As you advised, we revised the introduction to focus more on the association between physical function and hearing loss by revising some sentences and removing lengthy information for the effect of comorbidities such as hypertension and coronary artery disease. We summarized the gaps of previous studies looking at this association and described how this study address the gaps. (ll.72-73; ll.84-92)

**Comment 2:** Discussion. When comparing the current study with previous studies, it is important to compare the effect sizes of the associations and explain the differences.

**Response 2:** This is an important comment. We added a paragraph to describe about observed effect size and compare the differences with previous studies in discussion. (ll. 337-348)
